# Supplementary material for: Microwaves effectively examine the extent and type of coking over acid zeolite catalysts
Source: Nat Commun. 2017 Sep 11;8:514. doi: 10.1038/s41467-017-00602-8 (PMC5593951; doi:10.1038/s41467-017-00602-8)
Supplement: Supplementary file 1 — Supplementary Information [file 41467_2017_602_MOESM1_ESM.pdf]

Description of Supplementary Files

File Name: Supplementary Information
Description: Supplementary Figures, Supplementary Table, Supplementary Notes, Supplementary Discussion and Supplementary References

File Name: Peer Review File

Label in supplementary figures	Meanings
ZEO/ZEOLYST	Nano H-ZSM-5 zeolites from ZEOLYST company
COKE	Coked sample
SAPO34	H-SAPO-34 zeolite
MESO	Mesoporous H-ZSM-5 zeolite
MCM	H-MCM-22 zeolite
Ind	Industrial samples
46, 60, 160, 25, 20-30, 40-60	The Si/Al ratio of zeolite
2H/5H	2hour/5hour reaction time
TOP/BOT	Sample taken from the top/bottom of the catalyst bed

Supplementary Table 1 | Labels used in the figures and their meanings. Labels are used in the following Supplementary figures, and their meanings are listed in the Supplementary Table 1. In this research, we have tested a variety of zeolite types, with changes in the Si/Al ratio, and their particle size, or porosity. Differently coked samples are obtained by changing the reaction time, or taking from different positions in the catalyst bed.

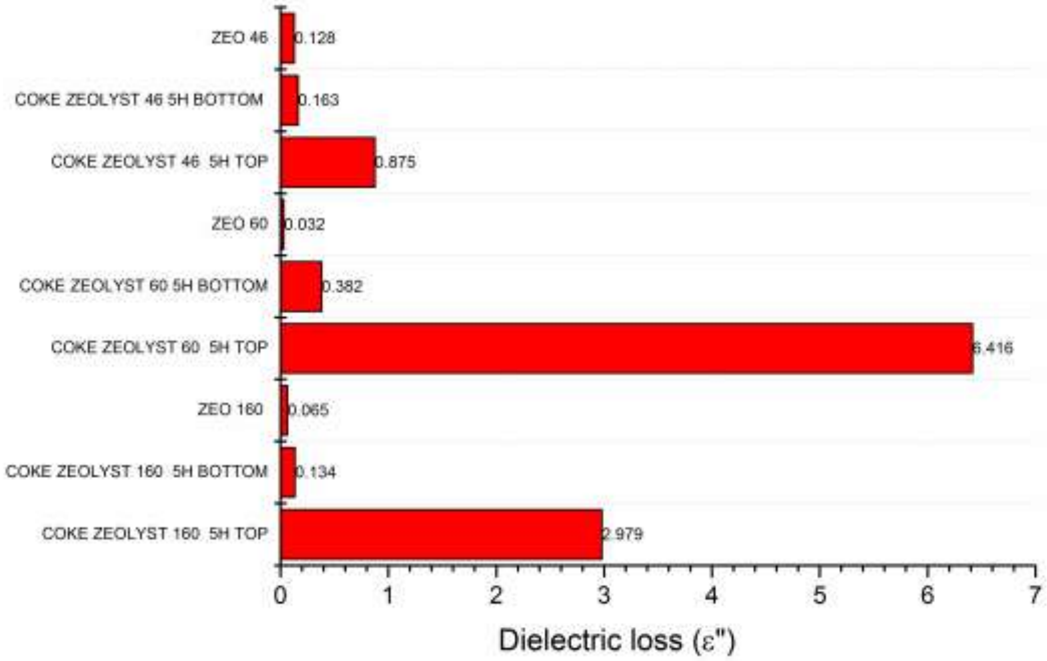

Supplementary Figure 1. Dielectric loss value (ε") of various nano zeolites, including the clean zeolite bodies, and the samples from the top and bottom parts of the catalyst bed with different coke depositions, the values were taken as an average of 5 times individual tests.

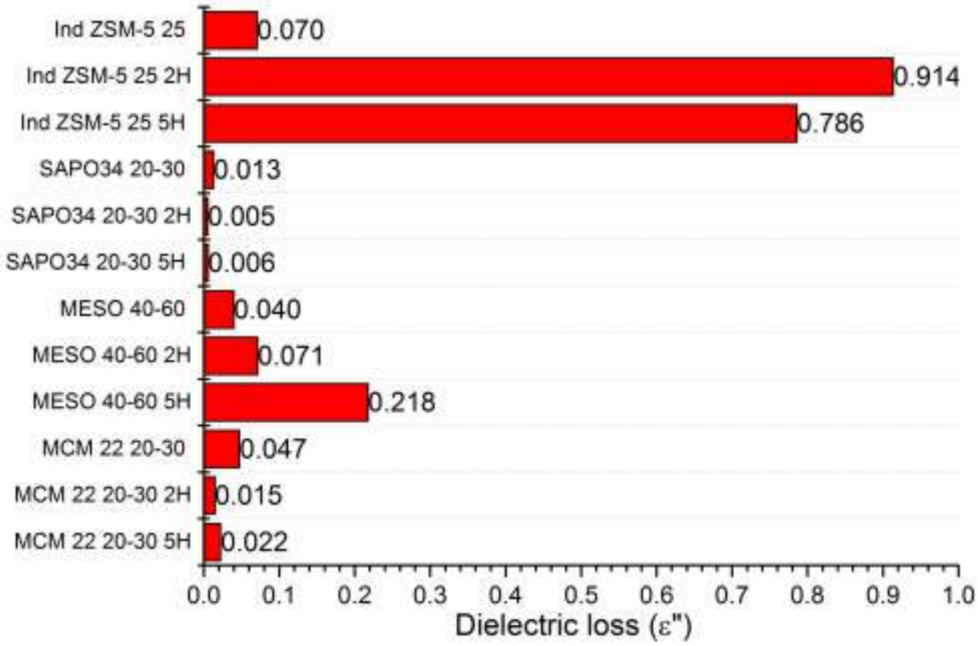

Supplementary Figure 2. Dielectric loss value (ε") of various zeolite types, including the clean zeolite bodies, and their 2h and 5h reacted samples with coke deposition, the values were taken as an average of 5 times individual tests.

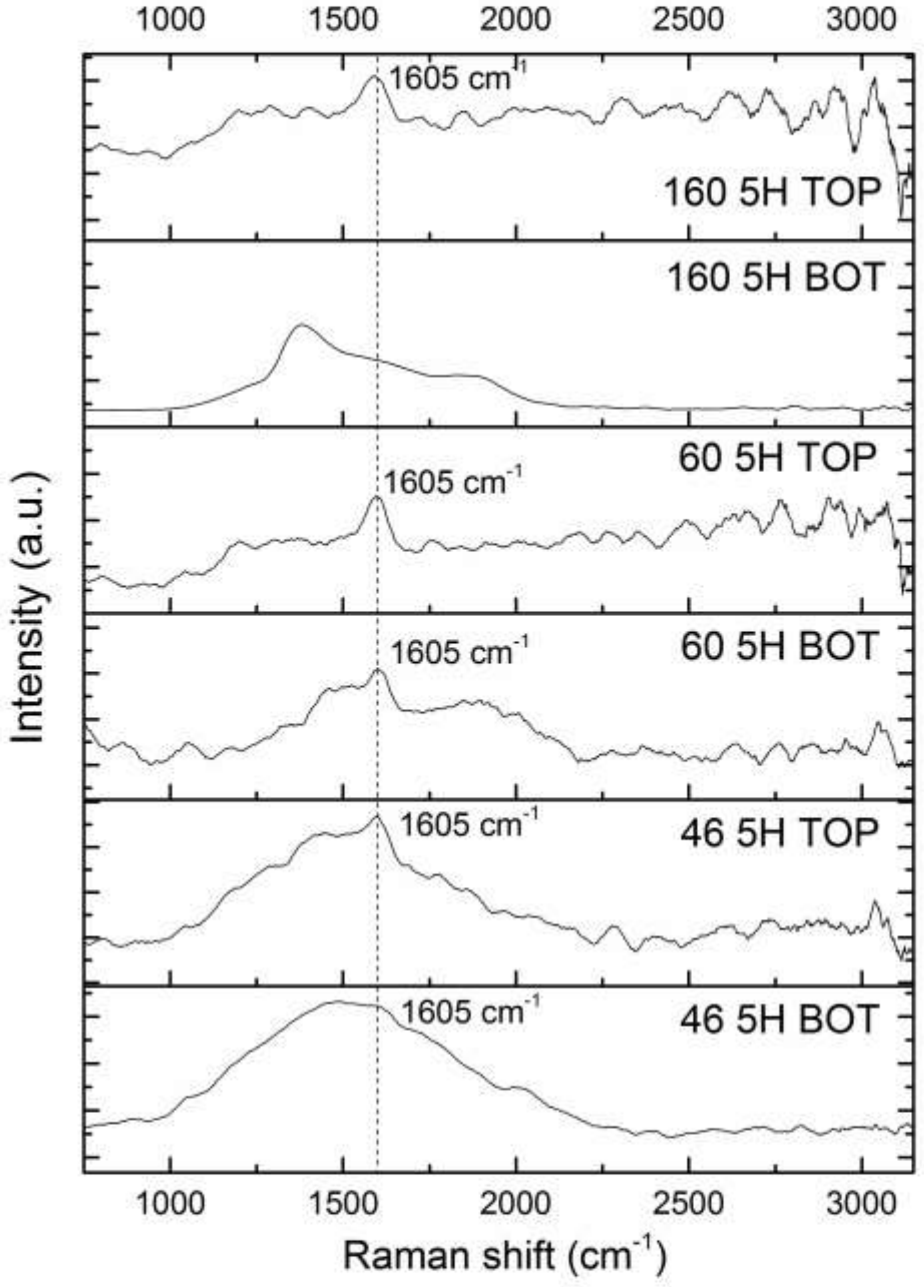

Supplementary Figure 3. Raman spectra of coked nano-ZSM-5 zeolites, for each sample the top and bot parts in the catalyst bed are separated.

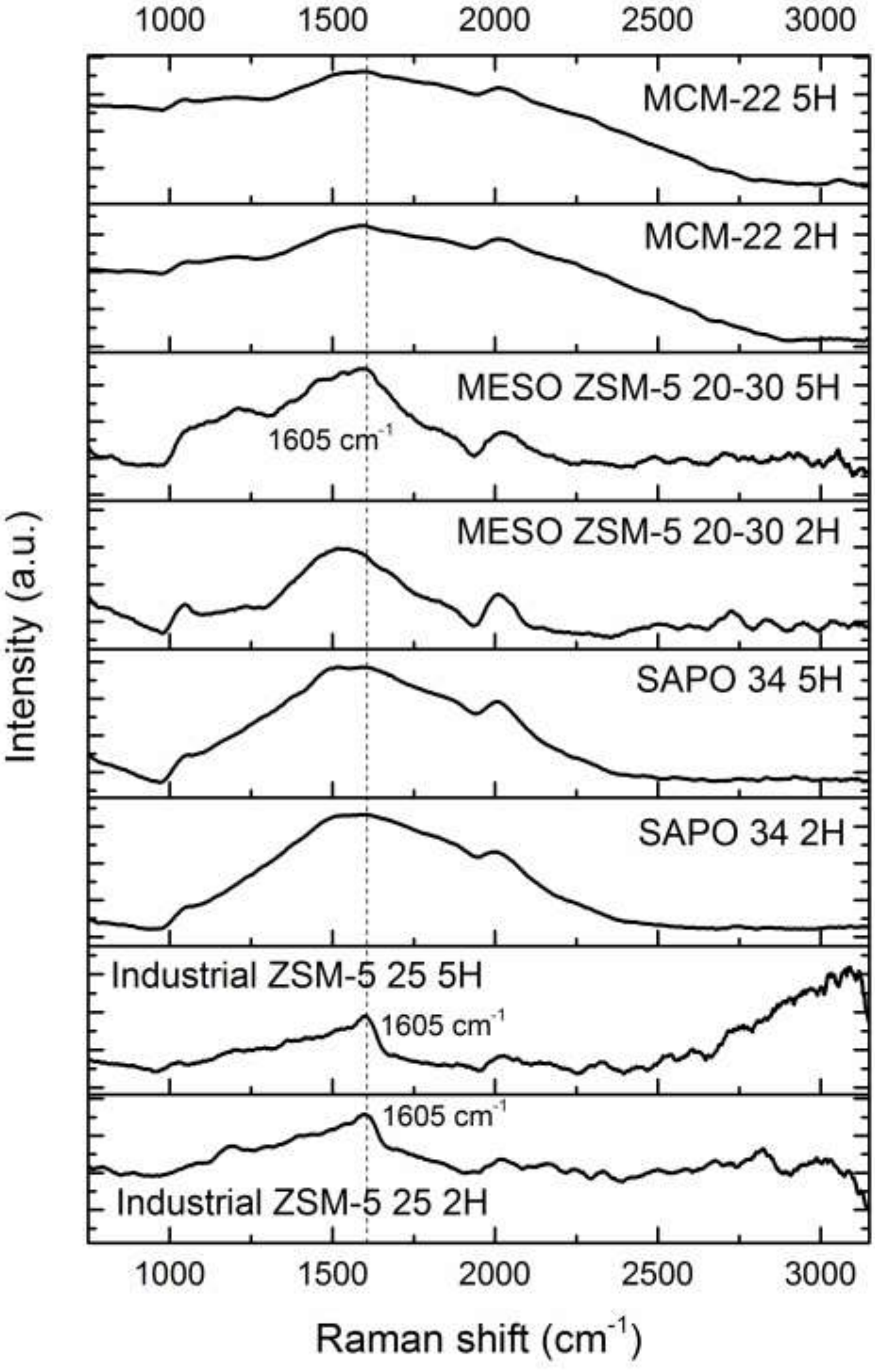

Supplementary Figure 4. Laser Raman spectra of various coked zeo-types after the MTH reaction (for each sample, reaction time varies between 2h and 5h).

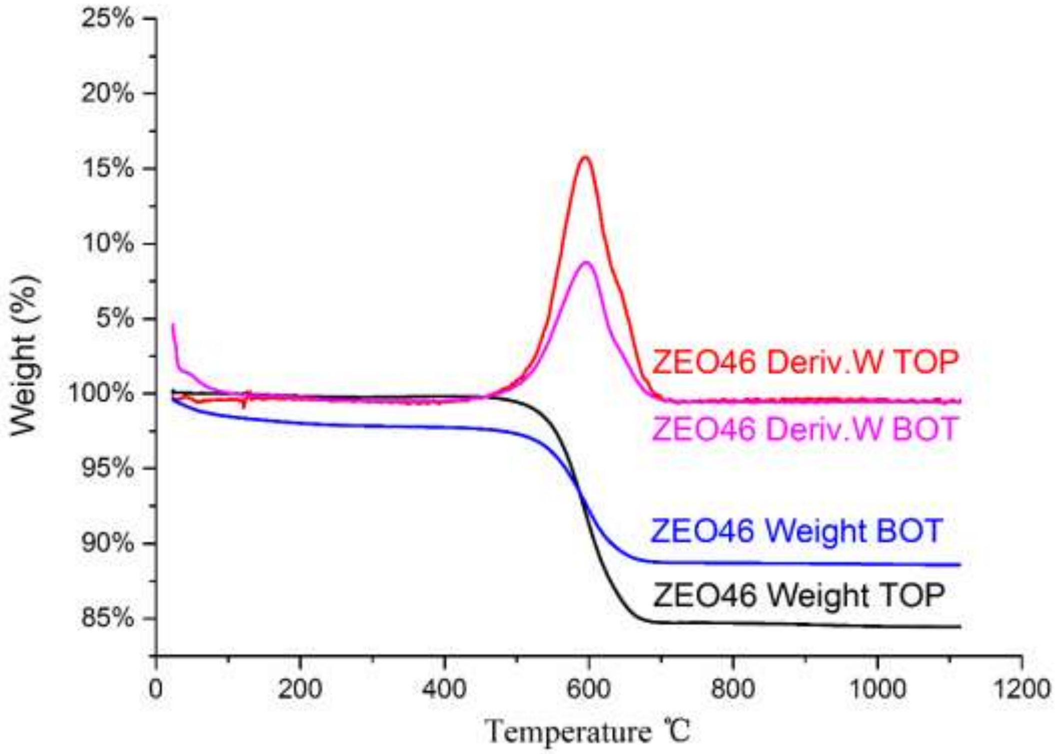

Supplementary Figure 5. Thermogravimetric analysis (TGA) plot of the post-reaction nano H-ZSM-5 (Si/Al=46) samples (in black and blue colors), and corresponding derivative thermogravimetry (DTG) curves (in red and purple colors).

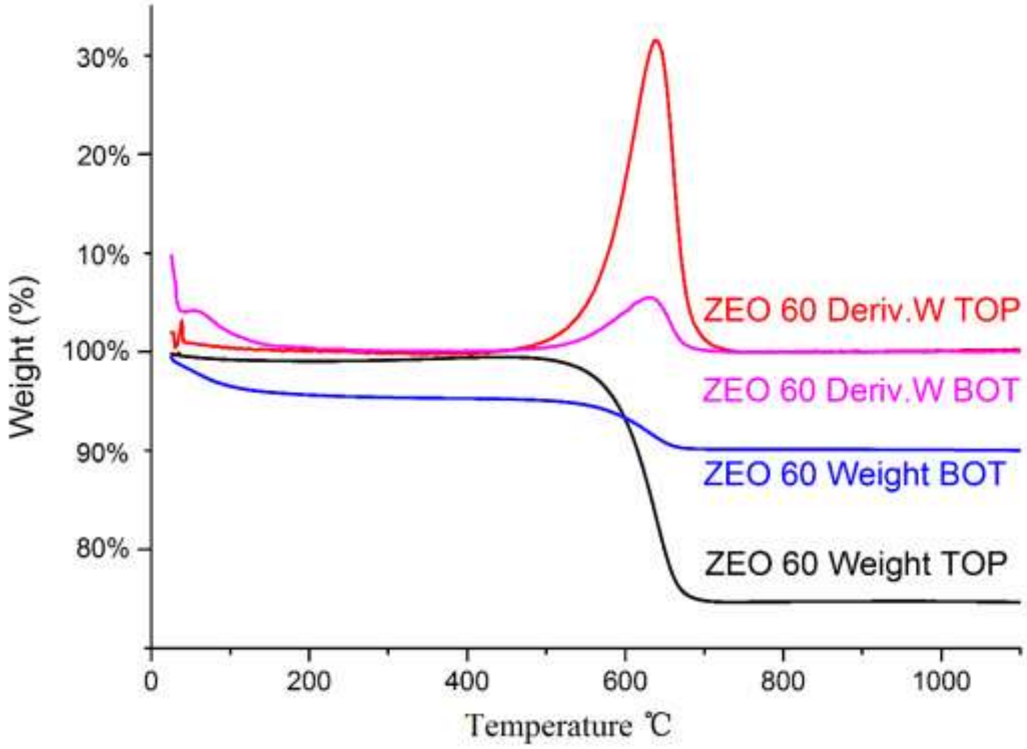

Supplementary Figure 6. Thermogravimetric analysis (TGA) plot of the post-reaction nano H-ZSM-5 (Si/Al=60) samples (in black and blue colors), and corresponding derivative thermogravimetry (DTG) curves (in red and purple colors).

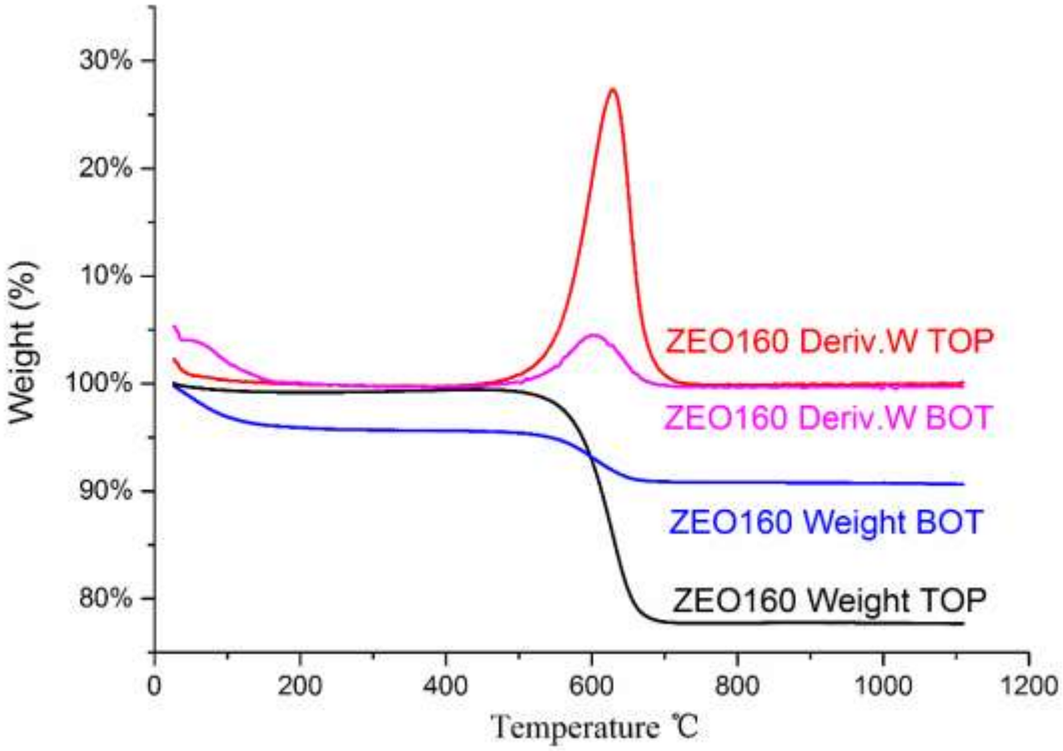

Supplementary Figure 7. Thermogravimetric analysis (TGA) plot of the post-reaction nano H-ZSM-5 (Si/Al=160) samples (in black and blue colors), and corresponding derivative thermogravimetry (DTG) curves (in red and purple colors).

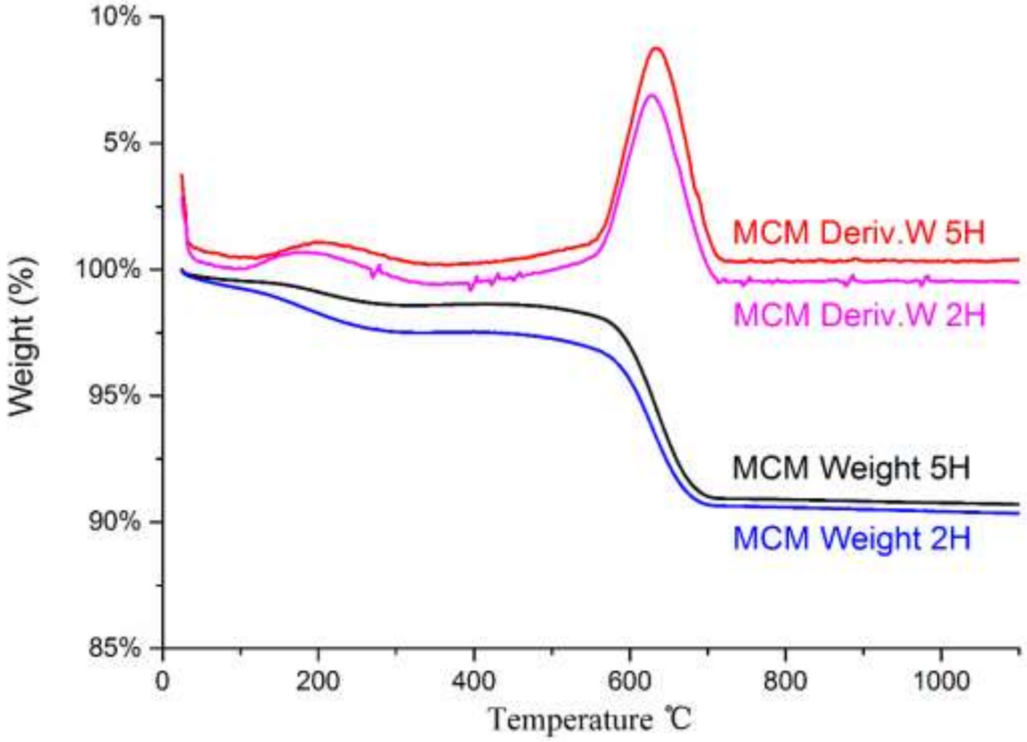

Supplementary Figure 8. Thermogravimetric analysis (TGA) plot of the post-reaction MCM (Si/Al=20-30) samples (in black and blue colors), and corresponding derivative thermogravimetry (DTG) curves (in red and purple colors).

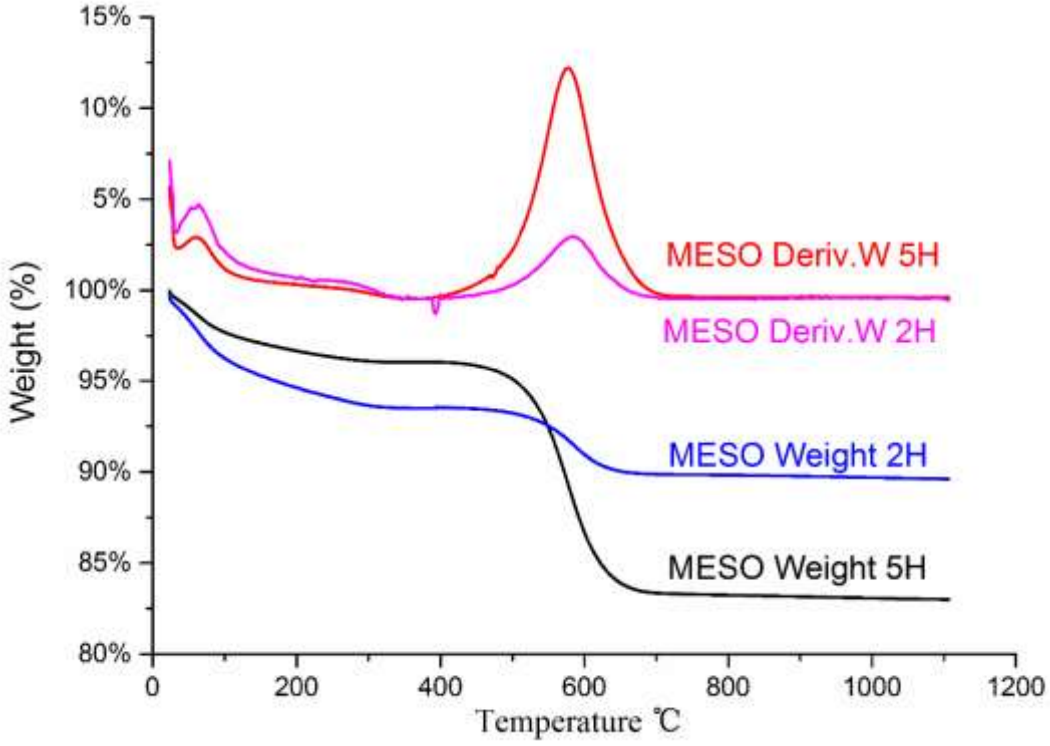

Supplementary Figure 9. Thermogravimetric analysis (TGA) plot of the post-reaction mesoporous H-ZSM-5 (Si/Al=40-60) samples (in black and blue colors), and corresponding derivative thermogravimetry (DTG) curves (in red and purple colors).

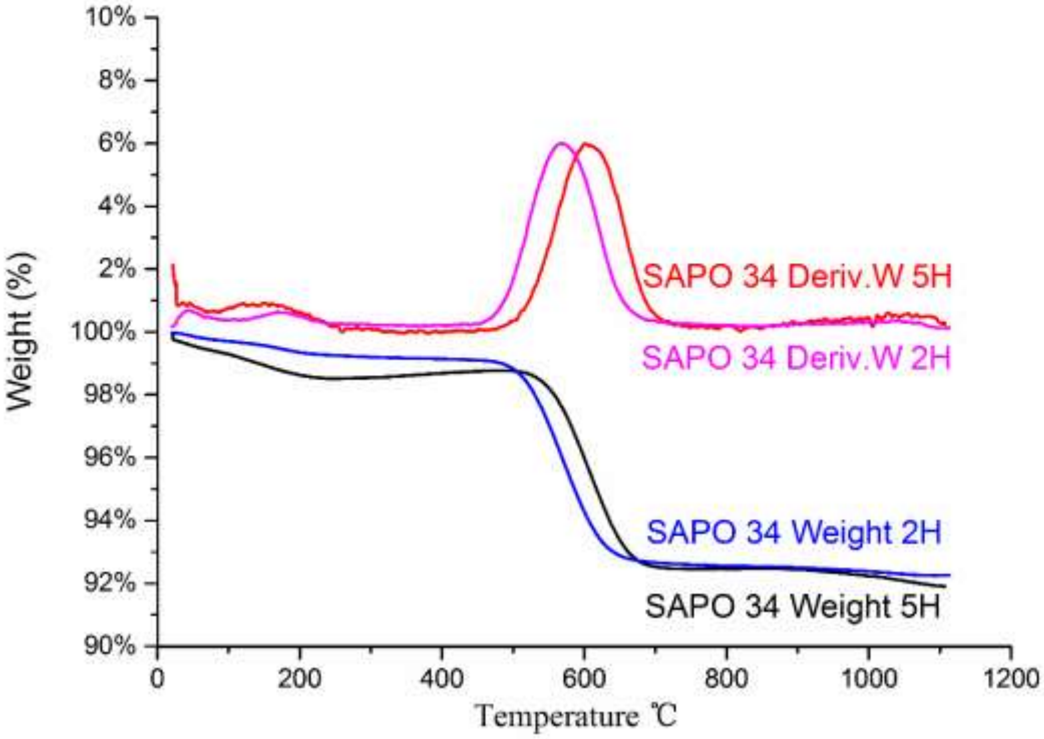

Supplementary Figure 10. Thermogravimetric analysis (TGA) plot of the post-reaction SAPO-34 (Si/Al=20-30) samples (in black and blue colors), and corresponding derivative thermogravimetry (DTG) curves (in red and purple colors).

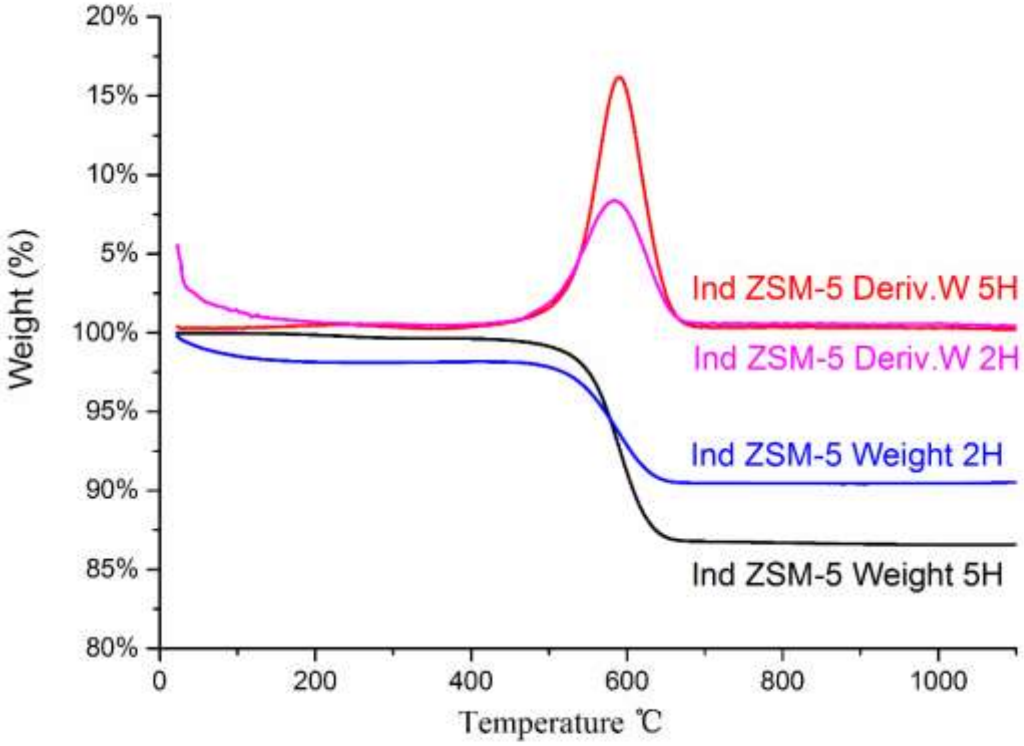

Supplementary Figure 11. Thermogravimetric analysis (TGA) plot of the post-reaction Industrial H-ZSM-5 (Si/Al=25) samples (in black and blue colors), and corresponding derivative thermogravimetry (DTG) curves (in red and purple colors).

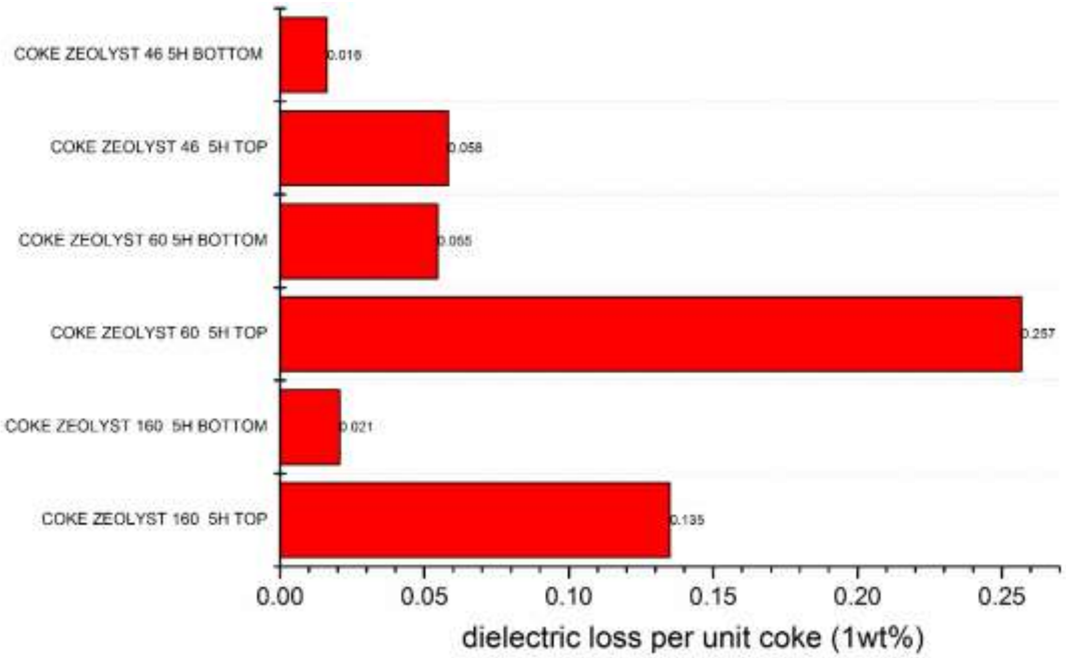

Supplementary Figure 12. Graph bar charts show normalized dielectric loss values (ε") by TGA weight loss (wt%) of each sample (coked nano H-ZSM-5 zeolites).

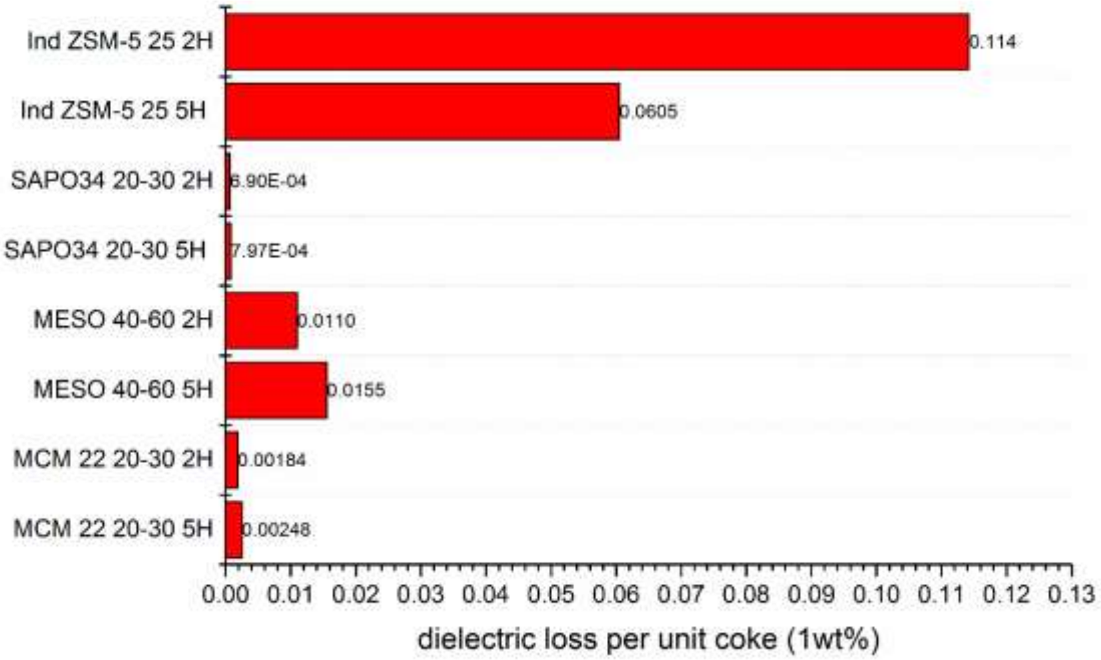

Supplementary Figure 13. Graph bar charts show normalized dielectric loss values (ε") by TGA weight loss (wt%) of each sample (multiple zeo-types).

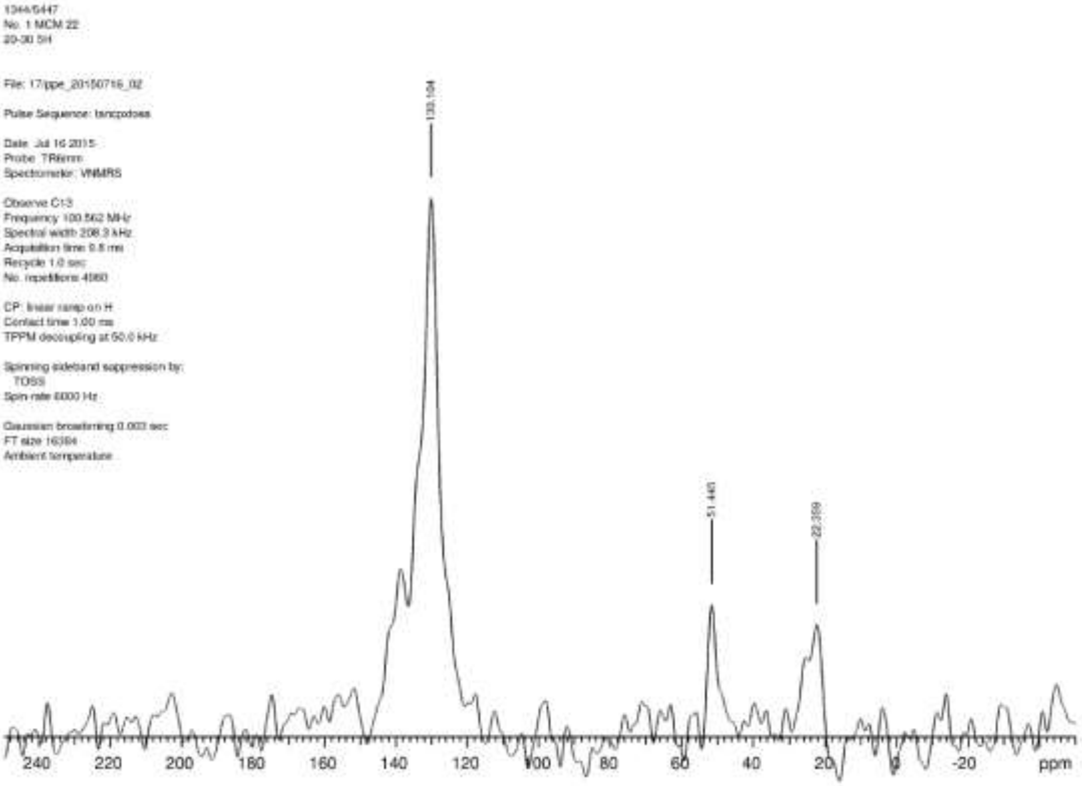

Supplementary Figure 14. ^{13}C NMR spectra of 5h reacted MCM-22 (Si/Al=20-30) showing coke composition.

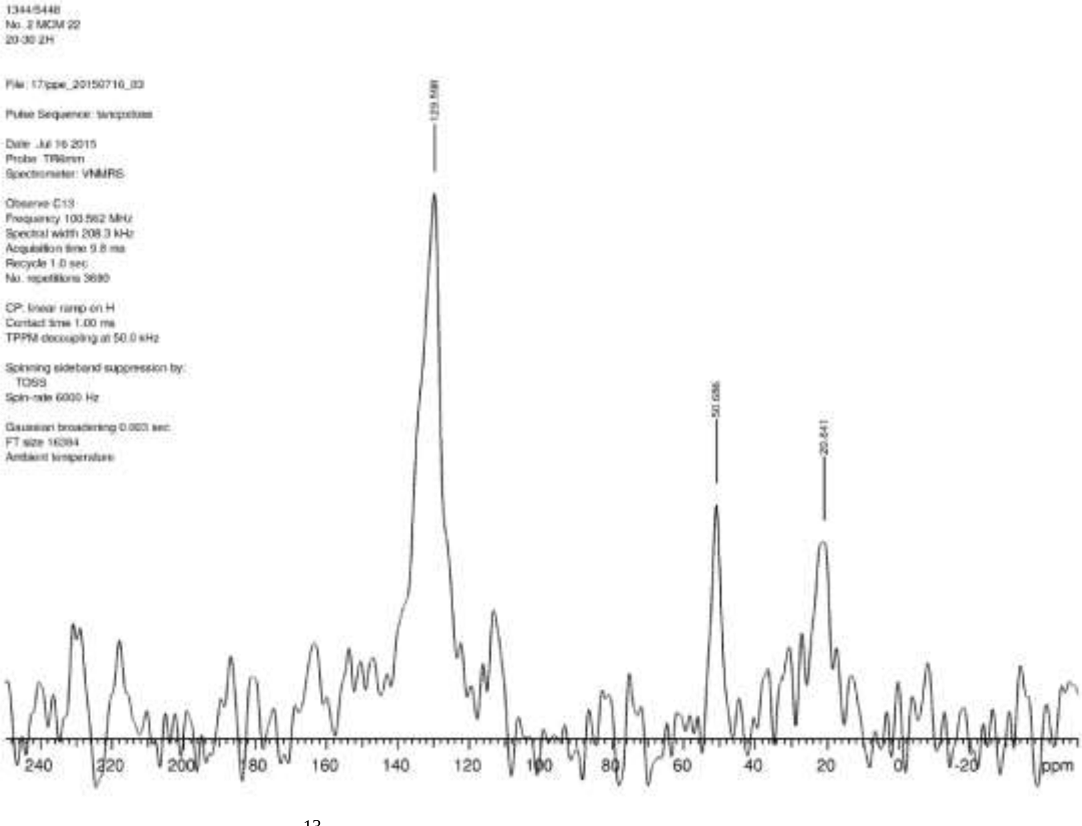

Supplementary Figure 15. ^{13}C NMR spectra of 2h reacted MCM-22 (Si/Al=20-30) showing coke composition.

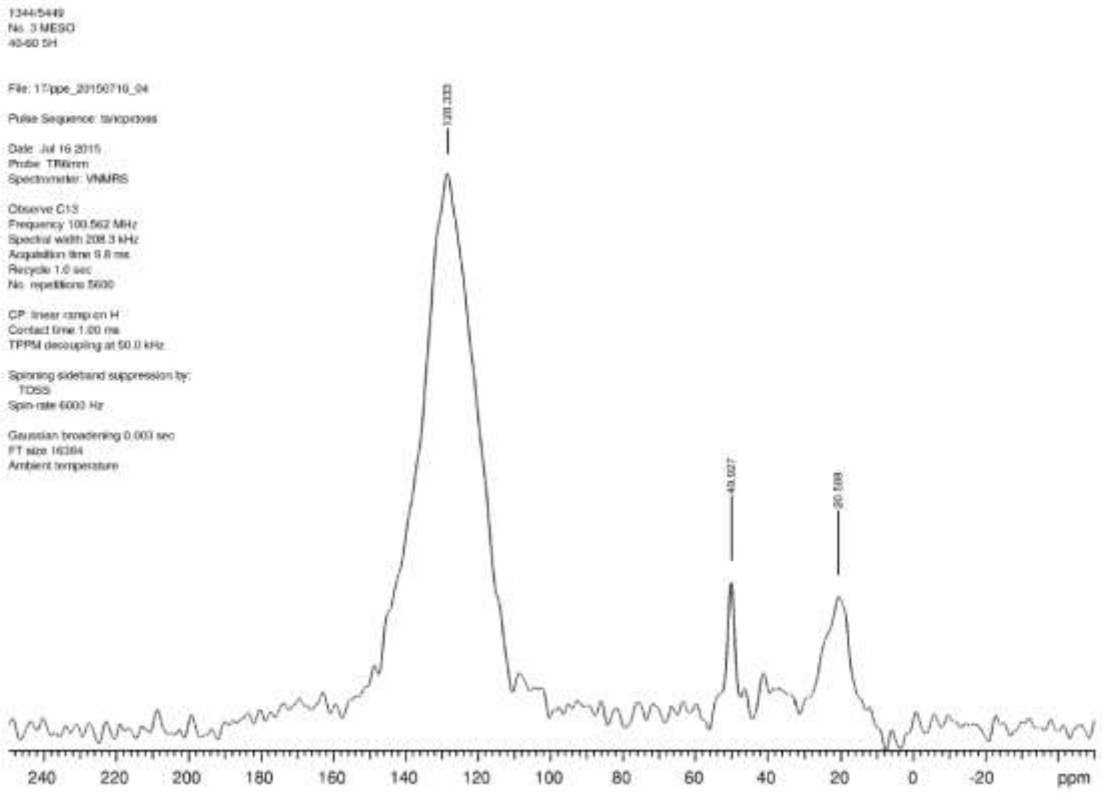

Supplementary Figure 16. ^{13}C NMR spectra of 5h reacted mesoporous ZSM-5 (Si/Al=40-60) showing coke composition.

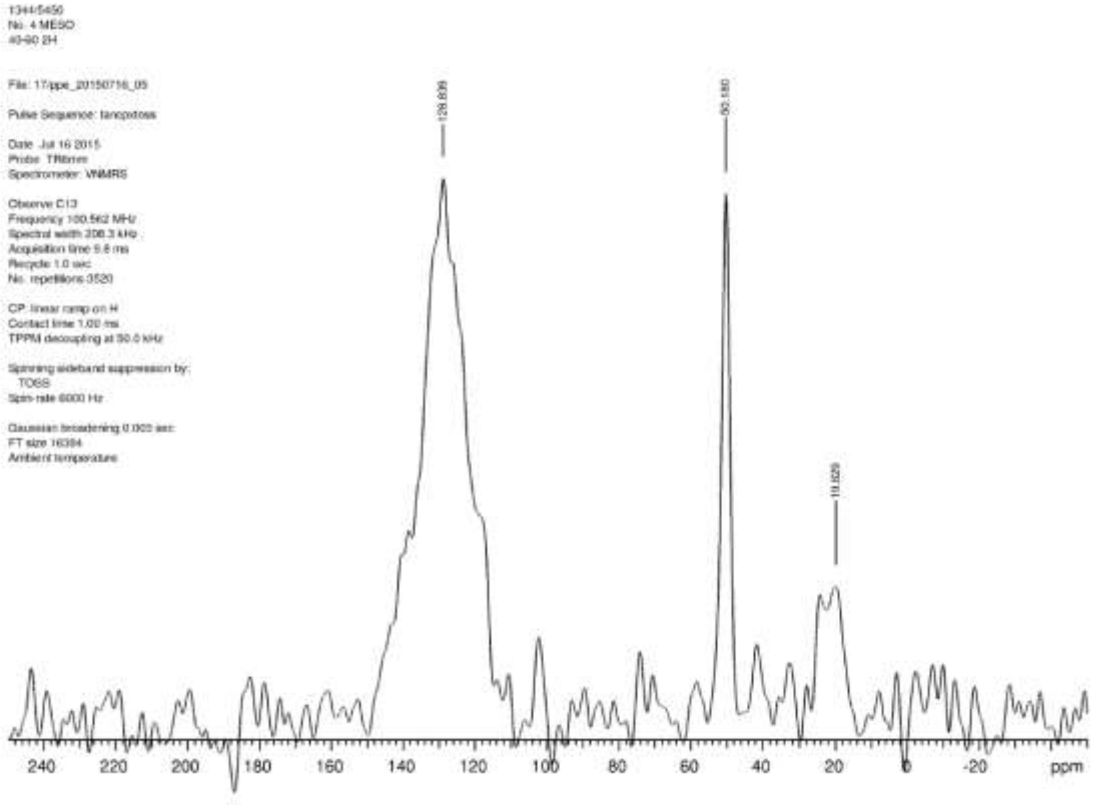

Supplementary Figure 17. ^{13}C NMR spectra of 2h reacted mesoporous ZSM-5 (Si/Al=40-60) showing coke composition.

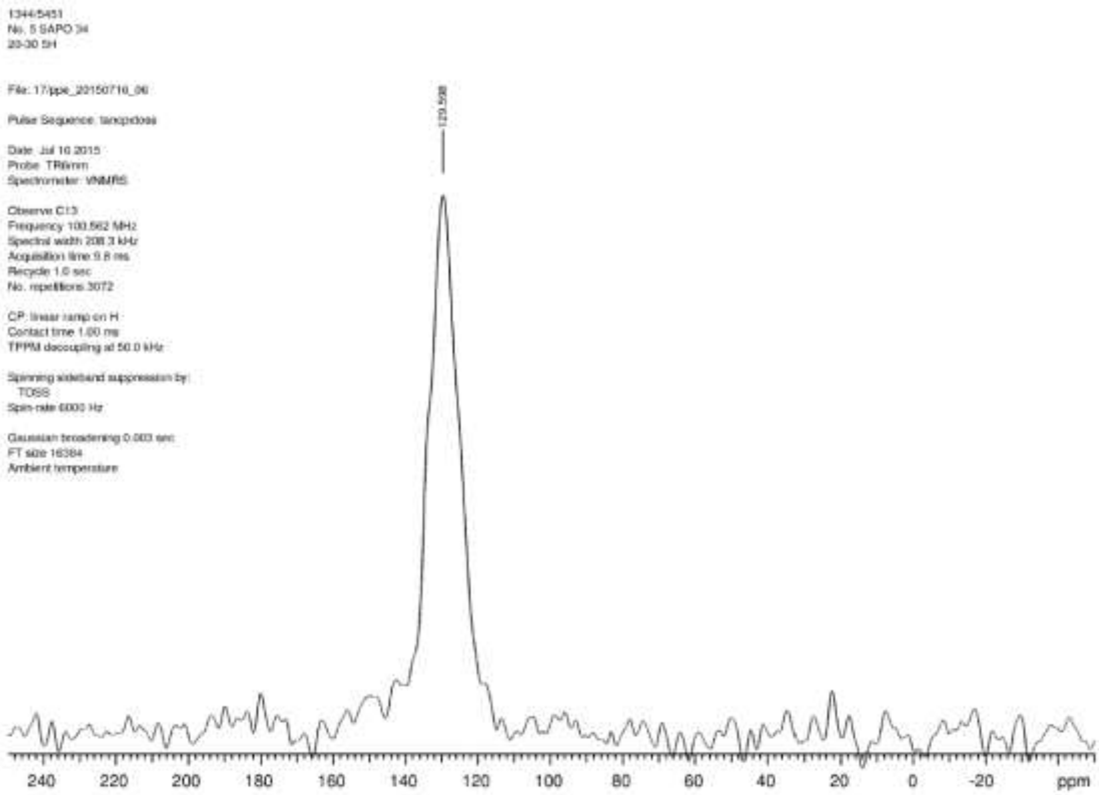

Supplementary Figure 18. ^{13}C NMR spectra of 5h reacted SAPO-34 (Si/Al=20-30) showing coke composition.

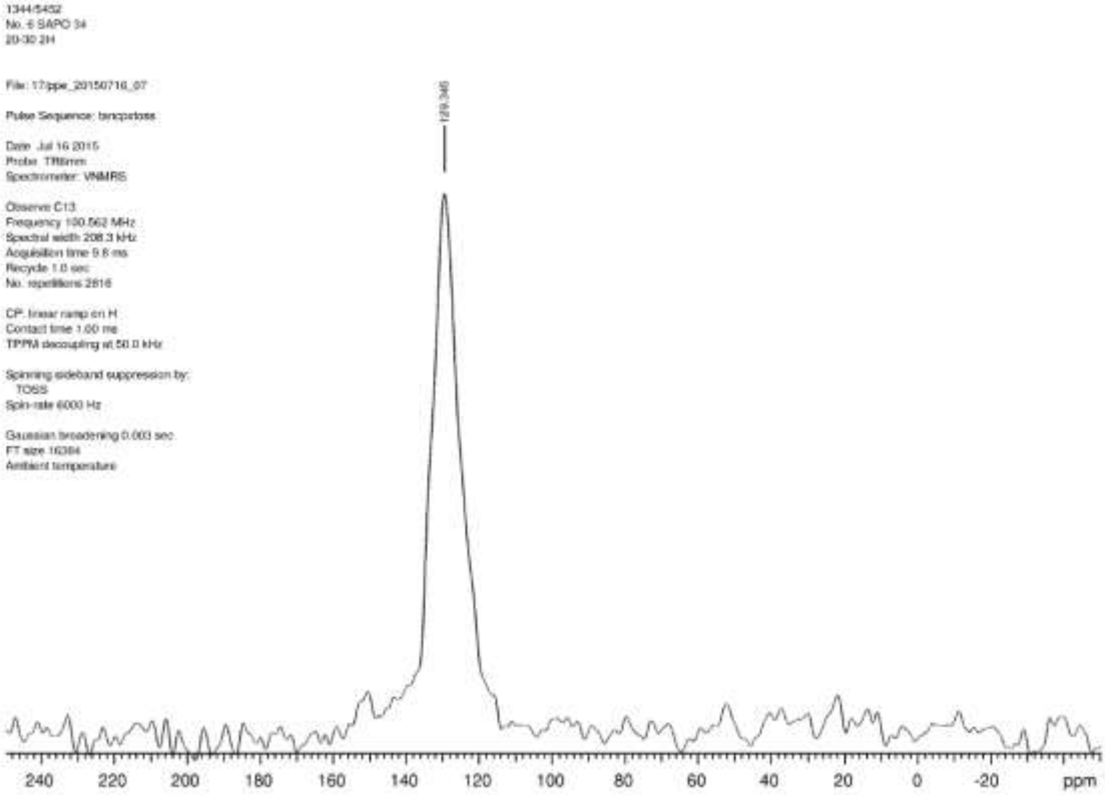

Supplementary Figure 19. ^{13}C NMR spectra of 2h reacted SAPO-34 (Si/Al=20-30) showing coke composition.

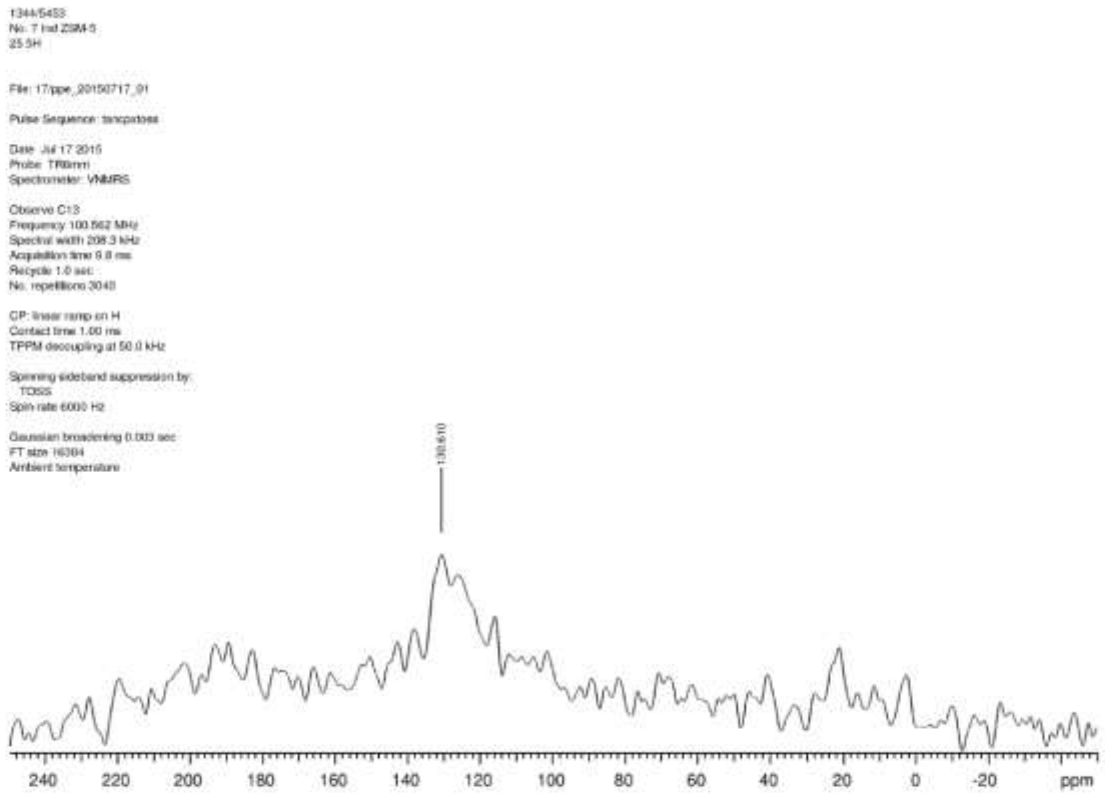

Supplementary Figure 20. ^{13}C NMR spectra of 5h reacted industrial ZSM-5 (Si/Al=25) showing coke composition

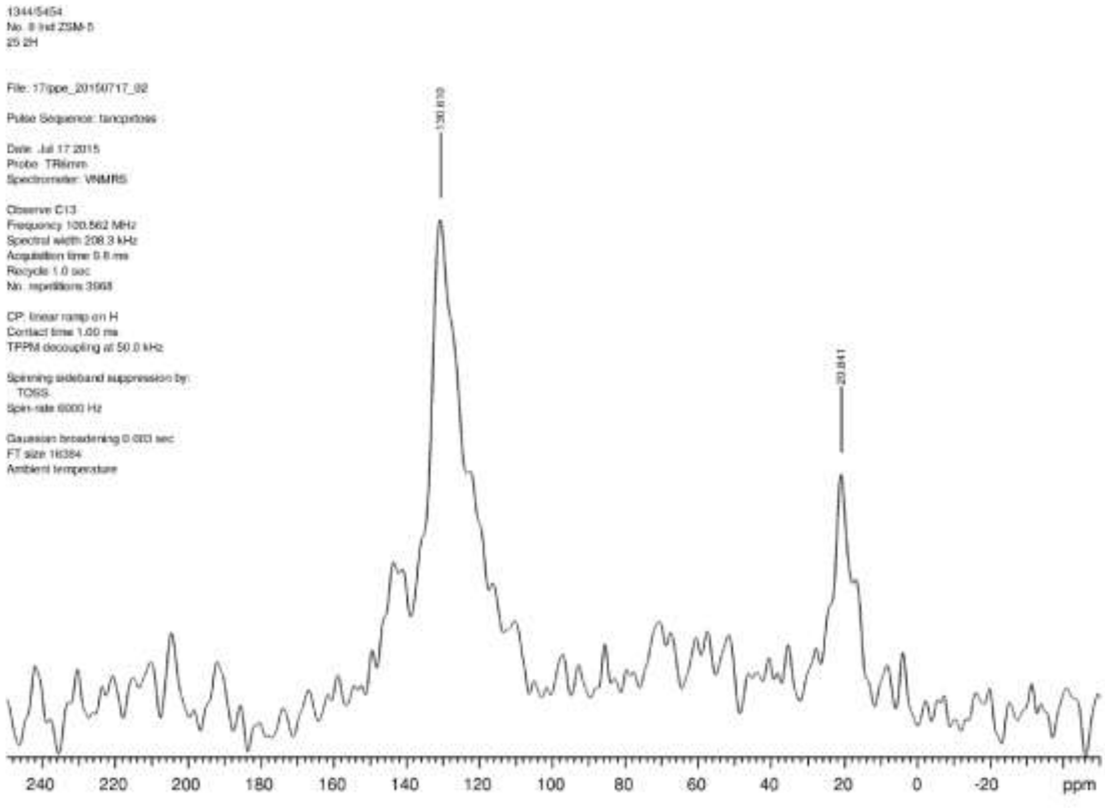

Supplementary Figure 21. ^{13}C NMR spectra of 2h reacted industrial ZSM-5 (Si/Al=25) showing coke composition

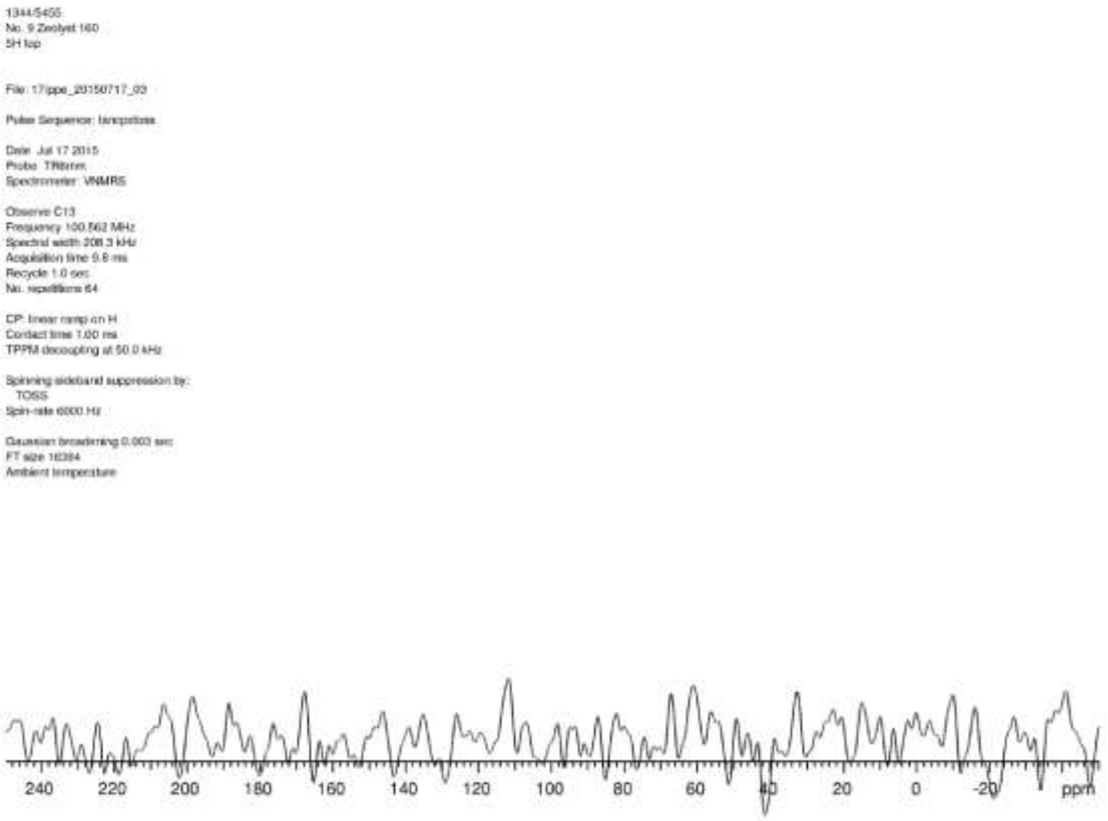

Supplementary Figure 22. ^{13}C NMR spectra of top-catalyst-bed coked nano ZSM-5 (Si/Al=160) showing coke composition.

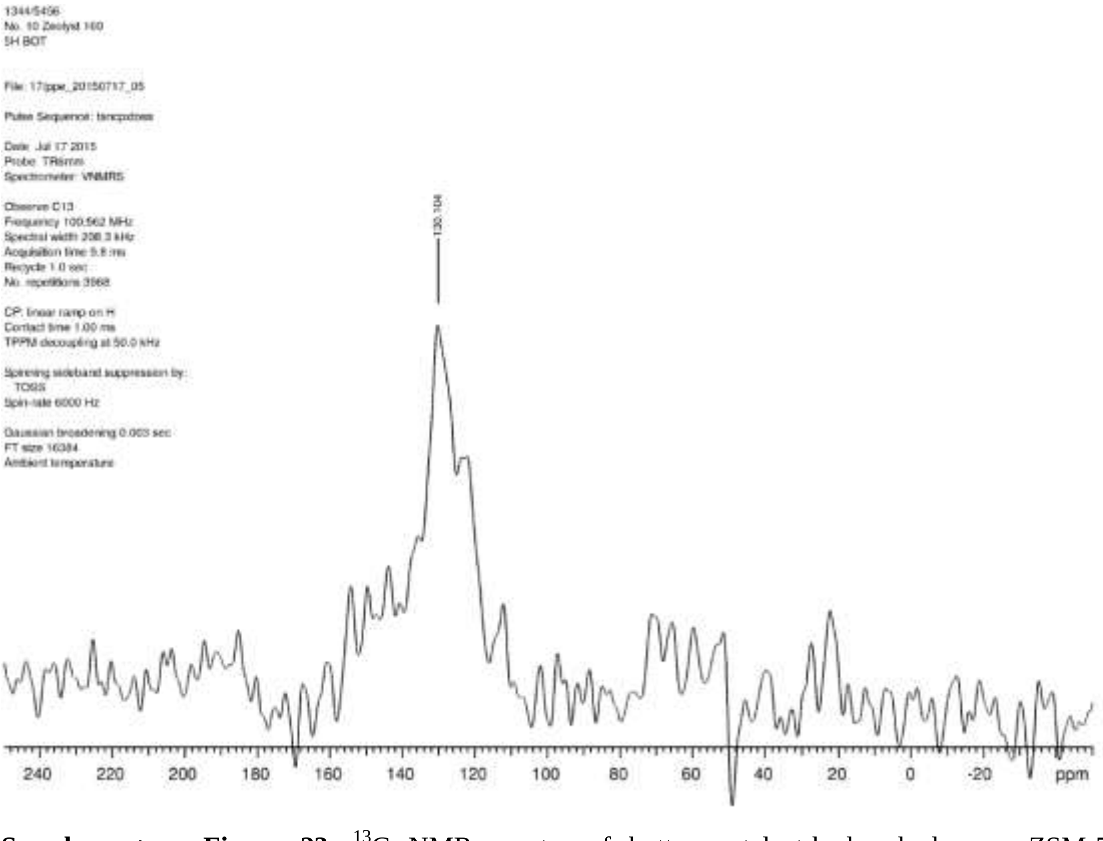

Supplementary Figure 23. ^{13}C NMR spectra of bottom-catalyst-bed coked nano ZSM-5 (Si/Al=160) showing coke composition.

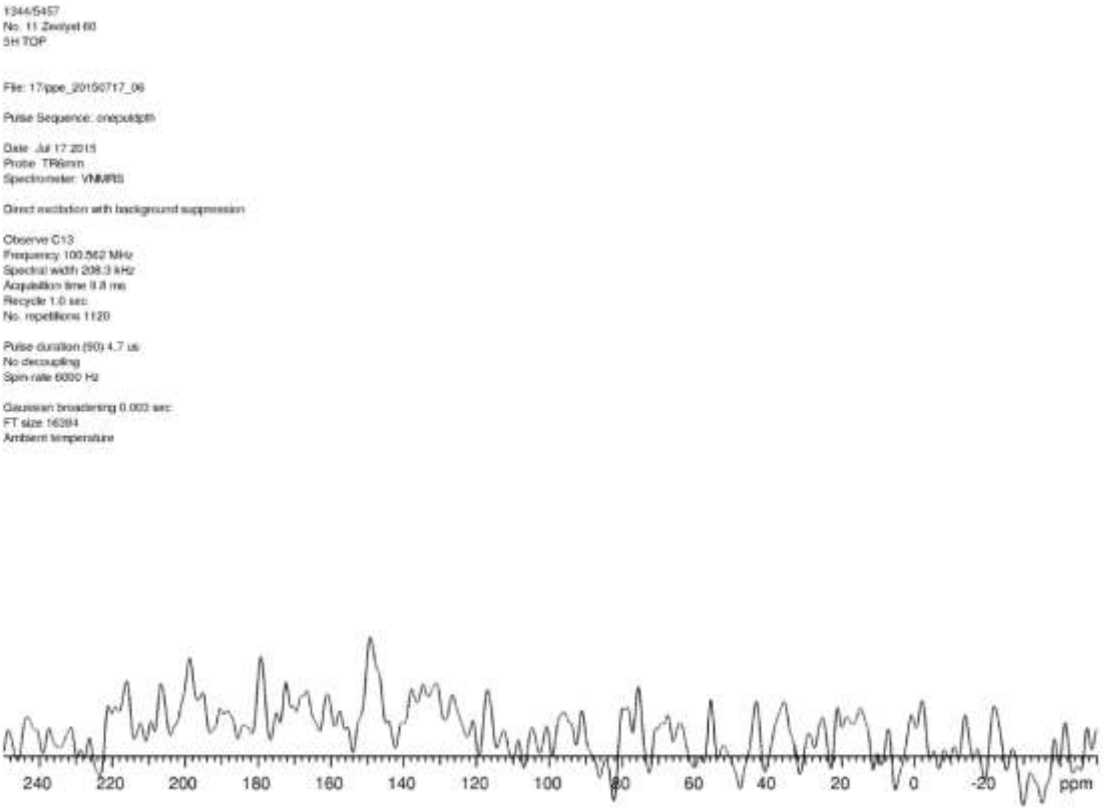

Supplementary Figure 24. ^{13}C NMR spectra of top-catalyst-bed coked nano ZSM-5 (Si/Al=60) showing coke composition.

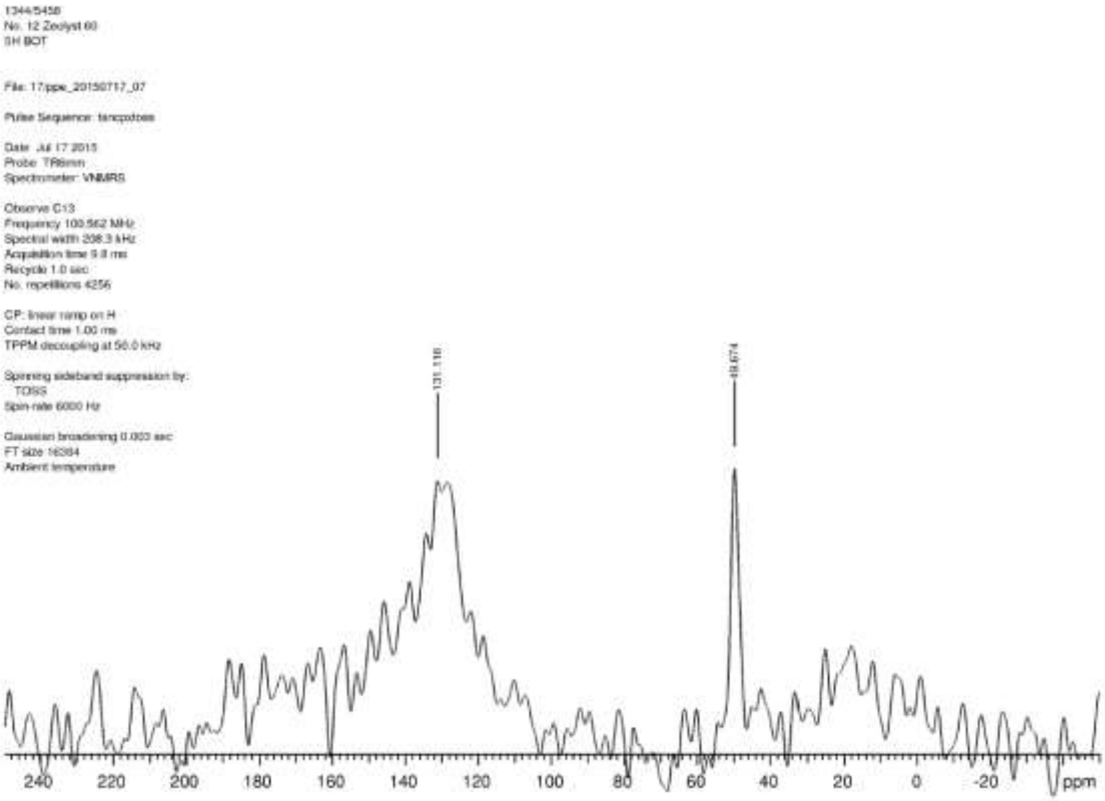

Supplementary Figure 25. ^{13}C NMR spectra of bottom-catalyst-bed coked nano ZSM-5 (Si/Al=60) showing coke composition.

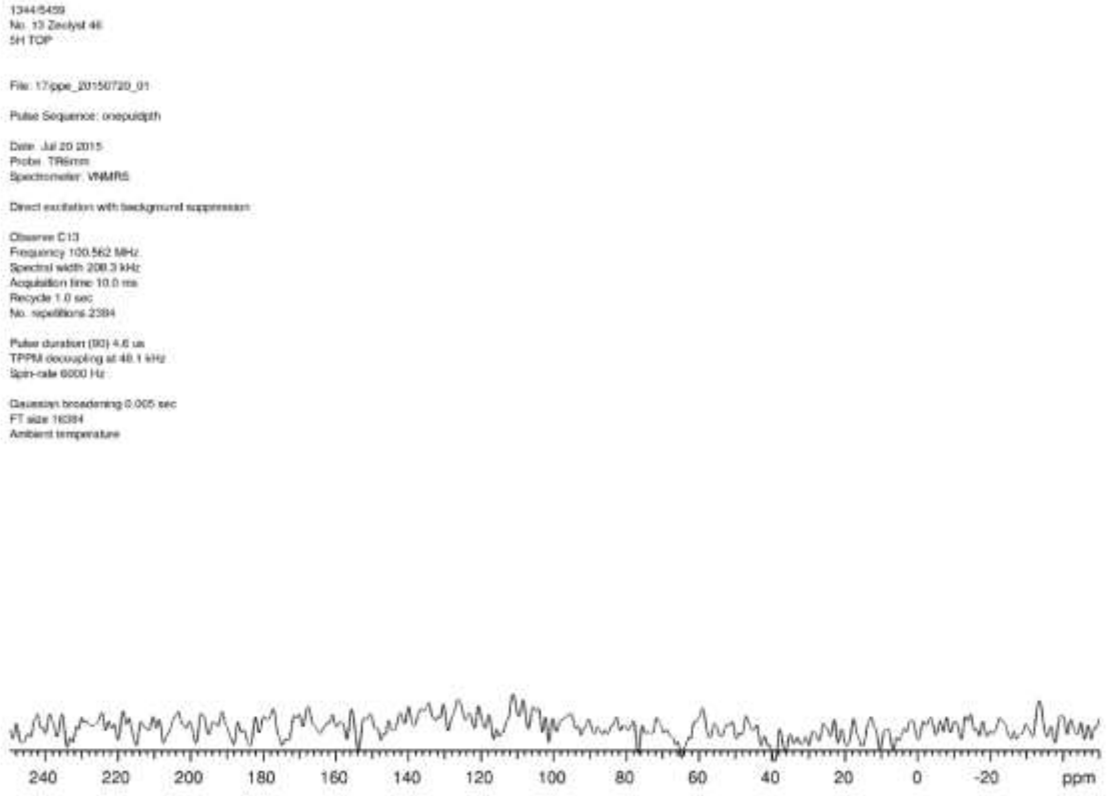

Supplementary Figure 26. ^{13}C NMR spectra of top-catalyst-bed coked nano ZSM-5 (Si/Al=46) showing coke composition.

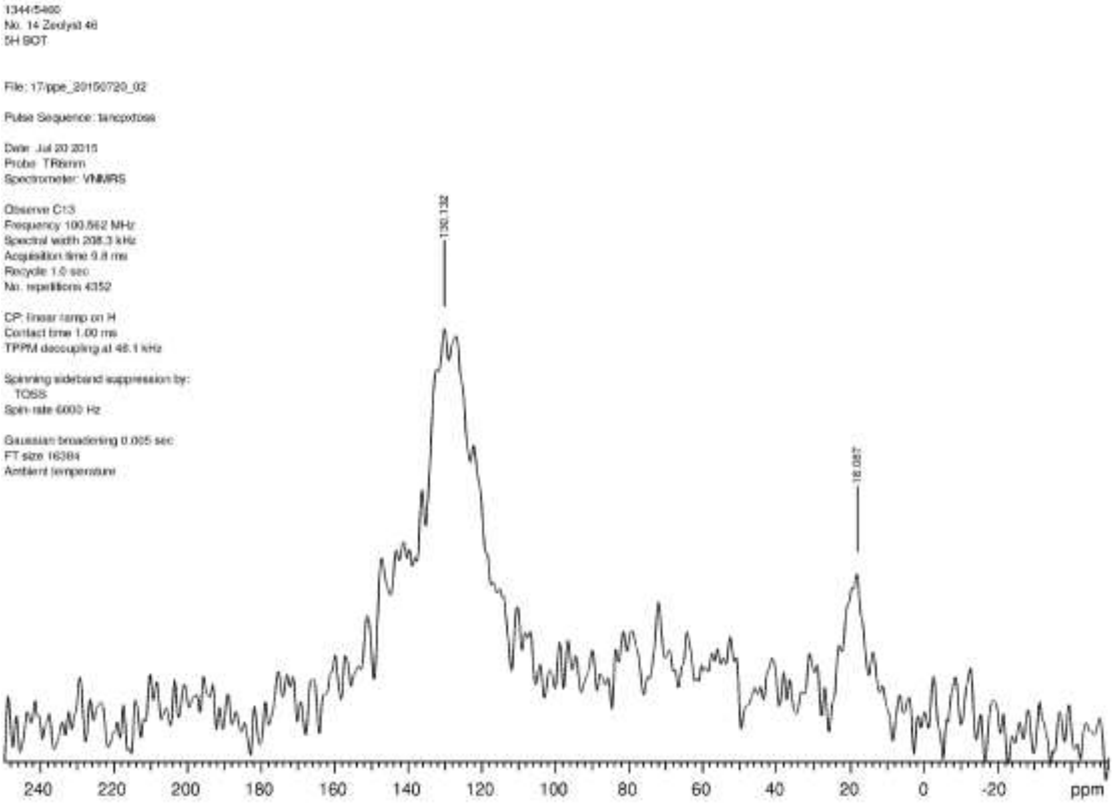

Supplementary Figure 27. ^{13}C NMR spectra of bottom-catalyst-bed coked nano ZSM-5 (Si/Al=46) showing coke composition.

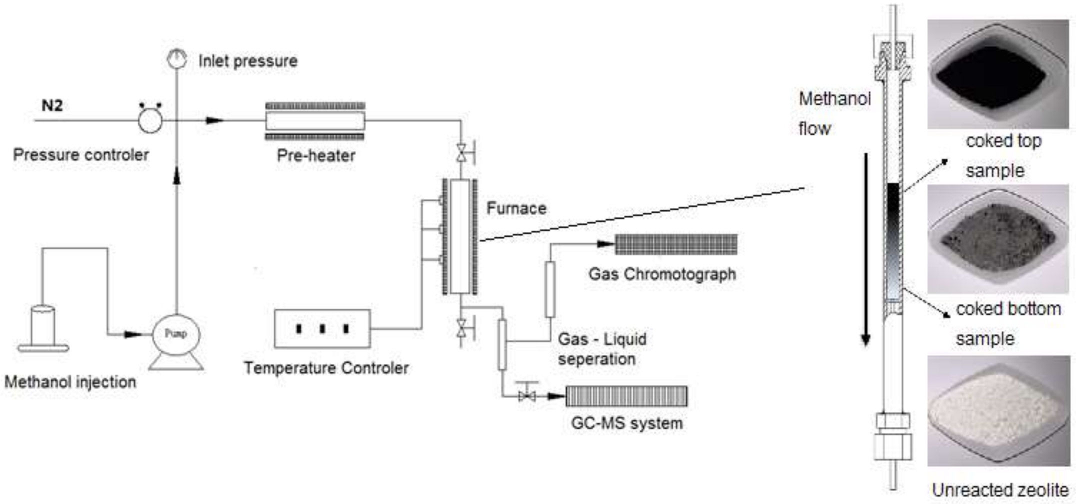

Supplementary Figure 28. Experimental system employed in the coking MTH reactions.

Supplementary Note 1. Here the measured ε” is an effective value affected by each sample constituent material (it is majorly but not all from the cokes, and not a simple adding up of the ε” values of each sample component as there are also interactions and other possible effects). The employed ε”/wt% value therefore reflects how unit weight cokes would affect the obtained, integral dielectric loss value of a coked sample, and it is not the ε” value of unit weight cokes.

Supplementary Note 2. It should be noted that the exact ε”/wt% values for most of the lightly-coked samples (e.g. the bottom or 2h reacted samples) should be lower than the currently presented data, as their TGA weight losses before 200 ^{o}C have not been taken into the calculation (this is because TGA weight loss due to moisture is also included in this temperature range), and there must have been a certain portion of coke weight accordingly missed due to the light organic deposits of lower volatile points (it seems that this is not the case for the heavily-coked samples with polyaromatics dominating in coke). Therefore, we would imagine that the real difference in signal response or the microwave absorption efficiency (i.e. ε”/wt%) between the heavily-coked samples (polyaromatics dominate) and lightly-coked samples (polyaromatics are not predominant) would be much more obvious than the current results.

Supplementary Note 3. The material should be dielectric, we consider the delimited electron distribution is crucial for effective electron frictions.

Supplementary Discussion

Measured all samples' ε", ε"/wt% and explanations. Supplementary Fig. 1 and 2 list the dielectric loss values (ε") of all coked samples and their parent zeolites. Corresponding coke compositions are illustrated in the obtained Laser-Raman spectra (Supplementary Fig. 3 and 4), where special attention has been paid to the bands at approximately 1605 cm^{-1} representing the polyaromatics in the coke contents (the exact assignment may vary in a very small range in different studies)[1-3]. The total coke contents are measured as weight loss in the Thermogravimetric analysis (TGA) of coked samples, as Supplementary Fig. 5-11 show (here the curves directly reflect the weight loss of sample in terms of wt%).

We further normalized the obtained dielectric loss value (ε") by weight of cokes for different post-run samples. Notably, the obtained result is not the ε" value of unit weight cokes (explanations in Supplementary Note 1), in fact, it indicates the contribution of unit weight cokes to the integral dielectric loss value of a coked sample, which is characteristic of the sample's coke composition. Data are presented in the form of ε"/wt%, as shown in Supplementary Fig. 12 and 13. Particularly, we directly employed the concentration (wt%) of coke contents in the post-run sample obtained from TGA measurement instead of a calculated coke weight in the sample tube. The total sample weight loaded in the tube and measured in the cavity changes in a quite small range between different samples by very careful loading operations; thus, the wt% value can be directly employed to represent the real coke weight measured, with negligible errors. The special format at the current stage, as we considered, makes the MW absorption data better combine with the TGA data (wt% of sample taken by cokes is directly readable form the TGA curves); on the other hand, the presented data also reflect how coke concentration in sample affects MW absorption at different coking levels, which possesses unique research significance for next-step studies (for instance, cokes taking 1wt% of the post-run sample contribute discrepantly to the integral sample dielectric loss value, between the cases that there are 10wt% and 20wt% of the post-run sample are cokes, respectively).

It should be noted that the exact ε"/wt% values of most of the lightly-coked samples (e.g. the bottom or 2h reacted samples, where polyaromatics are not predominant in total coke contents) should be lower than the currently presented data, as their TGA weight losses before 200 ^{o}C have not been taken into the calculation (explanations in Supplementary Note 2)[1]. All coked samples are protected in the N_2 flow after the reaction, and directly measured in TGA after very quick unloading at room temperature, so as the contamination by moisture in air can be reduced to the minimal level.

Discussion on ^{13}C NMR and other results. Coke compositions are further analyzed by ^{13}C NMR (Supplementary Fig. 14-27) with Cross Polarization (CP) and Direct Excitation (DE). The spectra of heavily-coked, polyaromatics rich samples (e.g. coked top-catalyst-bed nano H-ZSM-5 samples) show outstandingly poor ^{13}CP NMR signal, which indicates the over graphitized (dehydrogenated with more aromatic SP^2 carbons formed) status of these samples. This is a common problem of CP ^{13}C NMR when applied to measure samples containing a higher level of graphite-like carbons (the dissipation of hydrogen greatly limits the CP signals)[1]. Lightly-coked samples (e.g. coked bottom-catalyst-bed nano H-ZSM-5 samples) with poor aromatics in cokes and/or much less total coke amounts (these samples are visually shown in light black or grey colors), have shown somewhat more observable aromatic signals, at about 130ppm in the NMR spectra, in stark

contrast to their Raman and TGA results. The enhanced aromatic NMR signal here does not mean a rich abundance of aromatic cokes, however, it is as a result of a less-dehydrogenated sample status (hydrogen helps to improve the carbon signals) with in fact really poor amount of aromatics in total cokes.

The deeper graphitized status of those polyaromatics rich coked samples, as we proposed, is the most important reason for an enhanced ε''/wt% value. As is discussed in the main body of the paper, removal of hydrogen (a major effect in the coke formation) forms more aromatic sp^2 carbons in the coke structure. These sp^2 carbon centres possess further delocalized, and highly mobile π electrons which are able to undergo Maxwell-Wagner polarization to a greater extent and lead to the enhanced dielectric loss[4].

It should be noted that the pure zeolite body also possesses somewhat dielectric loss, and the obtained results in Supplementary Fig. 2 indicate that this dielectric loss property may be even stronger than the case that lightly-dehydrogenated coke species (aromatic cokes are not dominating) are deposited, as has been particularly observed on the samples of SAPO-34 and MCM-22. Although these samples possess somewhat more readable ^{13}C NMR signals from the aromatics (~130ppm), aromatics are still the rare components in total coke contents (the aromatic cokes will prohibit their CP ^{13}C NMR signals when their concentration increases, which happens to be a weakness of the ^{13}C NMR measurement without an ^{13}C exchange). On the other hand, these lightly-coked samples do not show any clear sign of polyaromatics in the corresponding Laser-Raman data, as shown in Supplementary Fig. 4, therefore we could imagine that their major coke contents are mainly non-aromatic based, might be some olefinic, or other paraffinic species deposited.

Here the discussion comes back to the question if we should subtract the dielectric loss value of pure zeolite body from the value of corresponding coked sample before the calculation of ε''/wt%, as part of the ε'' value should be given by the pure zeolite body. However, the measured permittivity of the sample is an effective value composed of complex contributions from each constituent material. The obtained data herein is not a simple adding up of ε'' values of each component, which is obviously not scientific. For instance, signals of NMR measurement which is a similar way also in sample-in-field mode are highly dependent on the surrounding environment of the nuclei (interactions and other effects caused by the sample composition also work on the final signal response), and therefore the results are not a simple adding up of responses from each sample component. In our microwave based measurement, there could also be potential interactions between the coke contents and the zeolite body, which affects the integral sample dielectric loss value.

Here the case of industrial H-ZSM-5 sample is even more complex. Its micro-sized, higher acid concentration (Si/Al=25) features often lead to rapid, bulky, and more complicated coke deposition. The 5h coked sample possesses some other condensed coke species represented by the bands extending towards to 3000 cm^{-1} in the Laser-Raman spectra, most possibly assigned to the poly-olefinic species (these are also hydrogen-loss carbonaceous species, therefore the 5h sample exhibits weak ^{13}C NMR signal)[1-3]. Accordingly, polyaromatics are not predominant in coke contents, and the corresponding dielectric loss value of this sample (5h coked industrial H-ZSM-5) is a bit smaller than the 2h coked sample which shows a dominating sign of polyaromatics in the Raman spectra. The special case of industrial H-ZSM-5 happens to present that, at the current stage, polyaromatics (a further graphitized structure than the condensed poly-olefinic species) in

coke contribute to the most effective microwave absorption (dielectric loss) compared with other species /coke precursors, most possibly owing to the higher abundance of sp^2 carbons in their structure, and particularly a further delocalized π electrons distribution (this is more efficient than olefins for Maxwell-Wagner Polarization). It seems that it is the evolution of carbon atoms from sp^3 hybridization (hydrogen saturated status) to a further electron delocalized sp^2 hybridization (dehydrogenated to graphite or graphite like status) allows us to monitor and assess the progressive catalyst coking. Such a process is just the nature of catalytic coke formation based on continuous, thermodynamically driven dehydrogenations. Particularly, this indeed arises our interests for a future, further exploration, with a final target on establishing a data base that combines the chemical feature of coke species with their particular MW absorption efficiencies (ε''/wt%).

Reactor settings and design of experiments. Supplementary Fig. 28 illustrates the fixed-bed-reactor system employed for Methanol-to-hydrocarbon reactions, with the experimental sets included in the main body of the paper (methods). The observed coke depositions are mainly attributed to the characteristic properties of different zeolites; however, the separated coking levels achieved by the same catalyst via different MTH reactions/conditions are based on series of long-term pre-explorations which helped to finally confirm the proper reaction approaches.

Supplementary References

1 Guisnet, M. & Ribeiro, R. F. *Deactivation and Regeneration of Zeolite Catalysts*. Vol. VOL.9 60-71 (Imperial College Press, 2011).

2 Chua, Y. T. & Stair, P. C. An ultraviolet Raman spectroscopic study of coke formation in methanol to hydrocarbons conversion over zeolite H-MFI. *J. Catal.* **213**, 39-46 (2003).

3 Li, C. & Stair, P. C. Ultraviolet Raman spectroscopy characterization of coke formation in zeolites. *Catalysis Today* **33**, 353-360 (1997).

4 Slocombe, D., Porch, A., Bustarret, E. & Williams, O. A. Microwave properties of nanodiamond particles. *Applied Physics Letters* **102**, 244102 (2013).

5 Porch, A., Slocombe, D. & Edwards, P. P. Microwave absorption in powders of small conducting particles for heating applications. *Physical Chemistry Chemical Physics* **15**, 2757-2763 (2013).
